# Whole-Genome Sequence of Burkholderia ambifaria Strain Q53, a Potential Plant Growth Promoter Isolated from the Rhizosphere of Peanut

**DOI:** 10.1128/mra.00021-23

**Published:** 2023-04-11

**Authors:** Fiorela Nievas, Santiago Revale, Emiliano Foresto, María Evangelina Carezzano, Emiliano Primo, Pedro Alzari, Mariano Martínez, Mathilde Ben-Assaya, Damien Mornico, Maricel Santoro, Walter Giordano, Pablo Bogino

**Affiliations:** a Instituto de Biotecnología Ambiental y Salud (INBIAS-CONICET), Departamento de Biología Molecular, Facultad de Ciencias Exactas, Físico-Químicas y Naturales, Universidad Nacional de Río Cuarto, Río Cuarto, Córdoba, Argentina; b Wellcome Centre for Human Genetics, University of Oxford, Oxford, United Kingdom; c Unité de Microbiologie Structurale, Institut Pasteur, CNRS UMR 3528, Université de Paris, Paris, France; d Hub de Bioinformatique et Biostatistique - Département Biologie Computationnelle, CNRS USR 3756, Institut Pasteur, Paris, France; e Department of Biochemistry, Max Planck for Chemical Ecology, Jena, Germany; University of Arizona

## Abstract

We report the complete genome sequence of Burkholderia ambifaria strain Q53, an environmental rhizobacterium isolated from the rhizosphere of peanut plants. The genome consists of 7.4 Mbp distributed into three circular chromosomes and was determined using a hybrid long- and short-read assembly approach.

## ANNOUNCEMENT

Members of the *Burkholderia* genus are versatile betaproteobacteria that inhabit diverse biotic and abiotic environments (1). They have a wide range of ecological roles (2, 3) which have attracted scientific interest due to their potential biotechnological applications (4).

Here, we announce the complete annotated genome of Burkholderia ambifaria Q53, a rhizospheric strain isolated from peanut roots. The plants were collected from an agricultural field in the south of the province of Córdoba, Argentina (33°31'38″S, 64°39'13″W). The collection of the rhizospheric soil and the isolation of the strain were carried out as described previously (5).

A pure culture of the strain was aerobically grown in LB medium (6) at 30°C until reaching the late exponential growth phase. This was the source for the total DNA, obtained with a DNeasy Blood & Tissue kit (Qiagen) for Illumina sequencing and a Promega Wizard HMW DNA extraction kit (Promega) for Oxford Nanopore Technologies sequencing. The genome was assembled through a hybrid approach including short Illumina reads and long Oxford Nanopore reads. The library was prepared with a Nextera XT DNA library preparation kit and sequenced on Illumina NextSeq 500, with a paired-end (PE) 150-bp read configuration. The sample was processed with an Oxford Nanopore Technologies rapid barcoding sequencing kit (SQK-RBK004) and a native barcoding genomic DNA sequencing kit (SQK-LSK109 with EXP-NBD104). The products of each were sequenced in two Flongle flow cells. Data were base called with Guppy v4.2.2, using the high-accuracy model and the --trim_barcodes option.

We obtained 5,397,708 Illumina PE reads and 43,449 Nanopore long reads (average of 4,981 bp, *N*_50_ of 9,460 bp), predicting 109-fold coverage and 29-fold coverage, respectively. Hybrid genome assembly was performed on the raw reads using the nf-core/bacass pipeline (commit ceebac0) with default parameters (7). This resulted in three contigs that were closed by manually analyzing the overlapped ends on Geneious software version 2019 2.1 (8). The reported genome consists of three chromosomes, which agrees with reports of several large replicons for this genus (9). The first chromosome had 3,495,354 bp, the second 2,728,612 bp, and the third 1,179,612 bp. Their G+C content was 66.8%, 66.6%, and 66.3%, respectively.

The complete genome, which was annotated using the NCBI Prokaryotic Genomes Annotation Pipeline (PGAP) (10–12), consists of 6,453 protein-coding sequences, six complete ribosomal operons, and 66 tRNAs.

As reported earlier for *B. ambifaria* (13, 14), the genome of Q53 harbors several genes related to plant growth promotion. For instance, there are genes predicted to encode the biosynthesis, transport, export, and iron uptake of siderophores, others predicted to be responsible for auxin biosynthesis, and yet others predicted to encode secondary metabolite biosynthesis, such as those related to antifungal activity. In addition, the genome has predicted genes associated with motility, biofilm development, and quorum sensing, all of which are typical of an environmental rhizospheric lifestyle. This complete sequence could be crucial for more in-depth research on the biological features of plant-associated *Burkholderia*.

### Data availability.

The complete genome sequence of *Burkholderia* sp. strain Q53 is available at NCBI GenBank under accession numbers CP092843.1, CP092844.1, and CP092845.1 with BioProject identifier (ID) PRJNA782308 and BioSample accession number SAMN23371909. Raw data reads are available at NCBI’s Sequence Read Archive under accession numbers SRR17038419 to SRR17038423.
